# Improving the temperature characteristics and catalytic efficiency of a mesophilic xylanase from *Aspergillus oryzae*, AoXyn11A, by iterative mutagenesis based on in silico design

**DOI:** 10.1186/s13568-017-0399-9

**Published:** 2017-05-15

**Authors:** Xue-Qing Li, Qin Wu, Die Hu, Rui Wang, Yan Liu, Min-Chen Wu, Jian-Fang Li

**Affiliations:** 1 School of Food Science and Technology, Jiangnan University, 1800 Lihu Road, Wuxi, 214122 China; 2 Key Laboratory of Carbohydrate Chemistry and Biotechnology, Ministry of Education, School of Biotechnology, Jiangnan University, 1800 Lihu Road, Wuxi, 214122 China; 3 Wuxi Medical School, Jiangnan University, 1800 Lihu Road, Wuxi, 214122 China

**Keywords:** Xylanase, Temperature characteristics, Catalytic efficiency, Site-saturation mutagenesis, Iterative mutagenesis, In silico design

## Abstract

To improve the temperature characteristics and catalytic efficiency of a glycoside hydrolase family (GHF) 11 xylanase from *Aspergillus oryzae* (AoXyn11A), its variants were predicted based on in silico design. Firstly, Gly^21^ with the maximum B-factor value, which was confirmed by molecular dynamics (MD) simulation on the three-dimensional structure of AoXyn11A, was subjected to site-saturation mutagenesis. Thus, one variant with the highest thermostability, AoXyn11A^G21I^, was selected from the mutagenesis library, *E. coli*/*Aoxyn11A* ^G21X^ (X: any one of 20 amino acids). Secondly, based on the primary structure multiple alignment of AoXyn11A with seven thermophilic GHF11 xylanases, AoXyn11A^Y13F^ or AoXyn11A^G21I–Y13F^, was designed by replacing Tyr^13^ in AoXyn11A or AoXyn11A^G21I^ with Phe. Finally, three variant-encoding genes, *Aoxyn11A* ^G21I^, *Aoxyn11A* ^Y13F^ and *Aoxyn11A* ^G21I–Y13F^, were constructed by two-stage whole-plasmid PCR method, and expressed in *Pichia pastoris* GS115, respectively. The temperature optimum (*T*_opt_) of recombinant (re) AoXyn11A^G21I–Y13F^ was 60 °C, being 5 °C higher than that of reAoXyn11A^G21I^ or reAoXyn11A^Y13F^, and 10 °C higher than that of reAoXyn11A. The thermal inactivation half-life (*t*_1/2_) of reAoXyn11A^G21I–Y13F^ at 50 °C was 240 min, being 40-, 3.4- and 2.5-fold longer than those of reAoXyn11A, reAoXyn11A^G21I^ and reAoXyn11A^Y13F^. The melting temperature (*T*_m_) values of reAoXyn11A, reAoXyn11A^G21I^, reAoXyn11A^Y13F^ and reAoXyn11A^G21I–Y13F^ were 52.3, 56.5, 58.6 and 61.3 °C, respectively. These findings indicated that the iterative mutagenesis of both Gly21Ile and Tyr13Phe improved the temperature characteristics of AoXyn11A in a synergistic mode. Besides those, the catalytic efficiency (*k*_cat_/*K*_m_) of reAoXyn11A^G21I–Y13F^ was 473.1 mL mg^−1^ s^−1^, which was 1.65-fold higher than that of reAoXyn11A.

**Electronic supplementary material:**

The online version of this article (doi:10.1186/s13568-017-0399-9) contains supplementary material, which is available to authorized users.

## Introduction

Xylanase (endo-β-1,4-d-xylanase, EC 3.2.1.8) exclusively catalyzes the hydrolysis of internal β-1,4-d-xylosidic linkages in the xylan backbone, producing xylooligosaccharides via a double displacement mechanism in which one conserved Glu operates as a general acid catalyst and the other Glu does as a nucleophile (Trevizano et al. 2012). Based on the multiple alignment of primary structures and the analysis of hydrophobic clusters, most of xylanases have been classified into glycoside hydrolase families (GHFs) 10 and 11 (Luo et al. 2010). The overall three-dimensional (3-D) structure of GHF11 xylanases resembles a partially closed right hand, consisting mainly of one α-helix and two β-sheets which are packed against each other (Xue et al. 2012). In striking contrast with GHF10 xylanases, the GHF11 counterparts possessed the catalytic activities merely towards d-xylose-containing substrates, low molecular weights (MWs <30 kDa), and alkaline isoelectric points (pIs >7.0) although some eukaryotic xylanases were acidic pIs (Balaa et al. 2009).

To date, xylanases have been applied to a range of industrial bioprocesses, such as feed, food, textile, biofuel and papermaking (Irfan et al. 2016). However, the majority of wild-type xylanases displayed low thermostability, preventing them from being applied in bioprocesses where the high temperature was required and encountered, exemplified by enzyme-added feeds, baking and pulp bleaching (Kumar et al. 2016). A handful of thermophilic xylanases have been produced by thermophiles, but their specific activities and other enzymatic properties were very poor, making them unable to be applied effectively (Zhang et al. 2014). To meet the increasing demands for thermophilic xylanases, some modifications in the primary and/or 3-D structures of mesophilic counterparts with superior enzymatic properties were conducted by peptide segment substitution, site-directed mutagenesis, DNA shuffling and error-prone PCR (Song et al. 2014; Stephens et al. 2014; Watanabe et al. 2016; Zheng et al. 2014). In recent years, various mutagenesis techniques have been developed for the modifications of proteins/enzymes. For example, the site-saturation mutagenesis of Glu^434^ on the surface of RGI lyase was carried out to improve its thermostability. After experimental verification, the best variant of RGI lyase, Glu434Leu, was selected from the mutagenesis library. Its half-life at 60 °C (*t*_1/2_^60^) extended to 31 min, which was 1.6-fold longer than that of the wild-type lyase (Silva et al. 2013). For the site-directed or site-saturation mutagenesis, the selection of the amino acid positions or the substituted residues was a pivotal step, which could be predicted based on in silico design or computer-aided design (Yin et al. 2015).

In our previous study, an AoXyn11A-encoding gene, *Aoxyn11A* (GenBank accession No. JQ326257), was expressed in *P. pastoris* GS115. The reAoXyn11A displayed the high specific activity, broad pH stability and strong tolerance to metal ions, but low thermostability (Li et al. 2013). In this work, to improve the temperature characteristics and catalytic efficiency of AoXyn11A, its variants were were predicted based on in silico design. Then, the variant genes, *Aoxyn11A* ^G21I^, *Aoxyn11A* ^Y13F^ and *Aoxyn11A* ^G21I–Y13F^, were constructed by site-saturation and site-directed mutagenesis, and expressed in *P. pastoris* GS115, respectively. Finally, the enzymatic properties of recombinant variants were analyzed, and compared to those of reAoXyn11A. To our knowledge, this is the first report on the saturation mutagenesis and high-throughput screening (Gly21Ile) and the iterative mutagenesis (Gly21Ile and Tyr13Phe) of AoXyn11A based on in silico design to improve its enzymatic properties.

## Materials and methods

### Strains, vectors, and culture media

*E. coli* BL21(DE3) and pET-28a(+) (Novagen, Madison, WI, USA) were used for the construction and screening of a site-saturation mutagenesis library, while *E. coli* DH5α and pPIC9K (Invitrogen, San Diego, CA, USA) for the construction of recombinant expression vectors. The vectors pET-28a-*Aoxyn11A* and pPIC9K-*Aoxyn11A*, and transformants *E. coli*/*Aoxyn11A* and *P. pastoris*/*Aoxyn11A* were constructed in our lab (Li et al. 2013). The genes, *Aoxyn11A* and its variants, were expressed in *P. pastoris* GS115. *E. coli* BL21 and DH5α were grown in the LB medium (10 g L^−1^ tryptone, 5 g L^−1^ yeast extract and 10 g L^−1^ NaCl, pH 7.2). *P. pastoris* and its transformants were cultured and induced in the YPD, MD, geneticin G418-containing YPD, BMGY and BMMY media, which were prepared as described in the manual of Multi-Copy *Pichia* Expression Kit (Invitrogen, USA).

### Multiple templates-based homology modeling

It was demonstrated that the validity of multiple templates-based modeling mainly relies on the primary structure identities of a target protein with template ones, and on the multiple alignment accuracy among crystal structures of templates (Madhusudhan et al. 2009). Compared to the single template-based modeling method, the multiple templates-based one greatly increased the facticity and accuracy of the modeled 3-D structure of a target protein (Sokkar et al. 2011). Therefore, using AoXyn11A primary structure as the template, homology sequences were searched among GHF11 xylanases at NCBI website (http://www.ncbi.nlm.nih.gov/) towards Protein Data Bank (PDB). The three known crystal structure xylanases separately from *Penicillium funiculosum* (PDB code: 1TE1) (Payan et al. 2004), *Talaromyces cellulolyticus* (3WP3) (Kataoka et al. 2014) and *E. coli* (2VUL) (Dumon et al. 2008), possessing the highest primary structure identities with AoXyn11A, were selected as homology modeling templates. The 3-D structures of AoXyn11A and its variants were modeled based on the three templates using the SALIGN program (http://salilab.org/DBAli/) and MODELLER 9.11 program (http://salilab.org/modeller/), and analyzed using the PyMol software (http://pymol.org/).

### Prediction of the three variants of AoXyn11A

The 3-D structure of a mesophilic protein/enzyme was more flexible than that of the corresponding thermophilic analog (Jaenicke and Böhm 1998). Therefore, to quantify the protein/enzyme flexibility, the notion of B-factor values was introduced to reflect the smearing of atomic electron densities as a result of thermal motion and positional disorder. B-factor values, namely atomic displacement parameters, of amino acids, were generated by molecular dynamics (MD) simulation on the crystal or modeled 3-D structure of protein using the GROMACS 4.5 package (http://www.gromacs.org/) and analyzed using the B-FITTER program (Reetz and Carballeira 2007). In this work, the modeled 3-D structure of AoXyn11A was subjected to MD simulation at 300 K for 15 ns, followed by calculating the B-factor values of residues. Gly^21^ in AoXyn11A with the maximum B-factor value was confirmed, and then randomly substituted by site-saturation mutagenesis.

AoXyn11A^Y13F^ or AoXyn11A^G21I–Y13F^ was designed by replacing Tyr^13^ in AoXyn11A or AoXyn11A^G21I^ with Phe, based on the multiple alignment of AoXyn11A with seven representative thermophilic GHF11 xylanases using the ClustalW2 program (http://www.ebi.ac.uk/Tools/msa/clustalw2/). Root mean square deviation (RMSD) value, which was defined as the C_α_-atomic displacement range of a protein from its original configuration to changed one at a high temperature and a certain time, was negatively correlative to its thermostability (Badieyan et al. 2012). Thereby, to predict the thermostabilities of AoXyn11A^Y13F^ and AoXyn11A^G21I–Y13F^, the modeled 3-D structures of AoXyn11A, AoXyn11A^Y13F^ and AoXyn11A^G21I–Y13F^ were subjected to MD simulations, respectively, at 500 K for 7 ns using the GROMACS 4.5 package, followed by calculating their RMSD values using a g_rms software in the GROMACS 4.5.

### Construction and screening of the site-saturation mutagenesis library

The site-saturation mutagenesis of a Gly^21^-encoding codon in *Aoxyn11A* into any amino acid-encoding codon was performed using the two-stage whole-plasmid PCR technique (Sanchis et al. 2008). The PCR primers used for gene mutagenesis were listed in Additional file 1: Table S1. The recombinant vectors, pET-28a-*Aoxyn11A* ^G21X^, were amplified from pET-28a-*Aoxyn11A*. In brief, the first-stage PCR amplification was carried out using a pair of PCR primers G21X-F (forward) and X11-R (reverse) under the following conditions: an initial denaturation at 98 °C for 3 min, followed by 30 cycles of at 98 °C for 10 s, 53 °C for 30 s and 72 °C for 45 s. Then, the second-stage PCR was performed using the first-stage PCR products as the primers: 30 cycles of at 98 °C for 10 s, 55 °C for 30 s and 72 °C for 5 min. The target PCR products (pET-28a-*Aoxyn11A* ^G21X^) were digested by *Dpn*I, and transformed into *E. coli* BL21, thereby constructing a site-saturation mutagenesis library (*E. coli*/*Aoxyn11A* ^G21X^).

Single colonies (*E. coli*/*Aoxyn11A* ^G21X^ strains) were separately inoculated into 500 μL LB medium containing 50 μg mL^−1^ kanamycin in a 96-well plate, and cultured at 37 °C overnight as the seed culture. Then, the same medium was inoculated with 2% seed culture, and grown until OD_600_ reached 0.6. Expression of *Aoxyn11A* ^G21X^ was induced by 1 mM IPTG at 28 °C for 8 h. The cells were collected, suspended in 100 μL Na_2_HPO_4_–citric acid buffer (50 mM, pH 5.5), and disrupted by ultrasonic (650 W, 180 cycles of work for 2 s and rest for 8 s). The resulting supernatant was divided into two aliquots and added into two 96-well plates. To screen the mutagenesis library, one plate was treated at 60 °C for 20 min, while the other plate used as the control. Then, aliquots of 5 μL treated or untreated supernatant were correspondingly added into two new plates with 95 μL 5.0 mg mL^−1^ xylan in each well, incubated at 50 °C for 10 min and stopped by adding 50 μL DNS reagent. Thus, the positive strains were selected, by which the expressed recombinant xylanases retained over 80% of their original activities, and in which the mutant genes were DNA-sequenced. Furthermore, one strain expressing reAoXyn11A^G21I^ with the highest thermostability, *E. coli*/*Aoxyn11A* ^G21I^, was obtained by extending treating time to 30 min at 60 °C.

### Site-directed mutagenesis of *Aoxyn11A* and *Aoxyn11A*^G21I^

The recombinant vector, pET-28a-*Aoxyn11A* ^G21I^, was extracted from the obtained strain *E. coli*/*Aoxyn11A* ^G21I^, and digested using *Eco*RI and *Not*I to release *Aoxyn11A* ^G21I^. Then, *Aoxyn11A* ^G21I^ was inserted into pPIC9K vector digested using the same enzymes, followed by transforming it into *E. coli* DH5α. The recombinant vector, pPIC9K-*Aoxyn11A* ^G21I^, was confirmed by DNA sequencing. Using pPIC9K-*Aoxyn11A* or -*Aoxyn11A* ^G21I^ as the template, the site-directed mutagenesis of Tyr^13^-encoding codon in *Aoxyn11A* or *Aoxyn11A* ^G21I^ into Phe-encoding codon was performed by two-stage whole-plasmid PCR using a pair of PCR primers Y13F-F (forward) and X11-R (reverse). The amplified target PCR products, that is, the recombinant vectors, pPIC9K-*Aoxyn11A* ^Y13F^ and -*Aoxyn11A* ^G21I–Y13F^, were digested using *Dpn*I, transformed into *E. coli* DH5α and confirmed by DNA sequencing.

### Transformation of recombinant vectors and screening of *P. pastoris* transformants

Three recombinant vectors, pPIC9K-*Aoxyn11A* ^G21I^, -*Aoxyn11A* ^Y13F^ and -*Aoxyn11A* ^G21I–Y13F^, were linearized with *Sal*I, and transformed into *P. pastoris* GS115, respectively, by electroporation using the Gene Pulser apparatus (Bio-Rad, Hercules, CA, USA). All *P. pastoris* transformants were primarily screened based on their abilities to grow on the MD plate, and then successively inoculated on the YPD plates containing G418 at concentrations of 1.0, 2.0 and 4.0 mg mL^−1^ for the screening of multiple copies of the integrated target gene. The *P. pastoris* transformant resisting high geneticin G418 concentration may have multiple copies of a gene, which can lead to the high expression of recombinant protein/enzyme (Invitrogen, USA). However, the expression level was not directly proportional to G418 concentration (Zhang et al. 2014). Thereby, a total of 60 *P. pastoris* transformants resistant to G418 of 4.0 mg mL^−1^, separately containing *Aoxyn11A* ^G21I^, *Aoxyn11A* ^Y13F^ and *Aoxyn11A* ^G21I–Y13F^, were picked out for expression tests.

### Expression and purification of the recombinant xylanases

Expression of *Aoxyn11A* ^G21I^, *Aoxyn11A* ^Y13F^ or *Aoxyn11A* ^G21I–Y13F^ in *P. pastoris* GS115 was performed according to the instruction of Multi-Copy *Pichia* Expression Kit (Invitrogen, USA) with slight modification. In brief, each single colony of 60 *pastoris* transformants was inoculated into 30 mL BMGY medium and cultured at 30 °C with 220 rpm until OD_600_ reached 2–4. Then, the cells were harvested by centrifugation, suspended in 30 mL BMMY medium and induced for the expression of recombinant xylanase by adding methanol to a final concentration of 1.0% at 24 h intervals at 30 °C for 72 h.

After expression, 10 mL supernatant containing the recombinant xylanase with a 6-His-tag at its C-terminus was loaded onto a nickel–nitrilotriacetic acid (Ni–NTA) column (Tiandz, Beijing, China; 1 × 6 cm) equilibrated with buffer A (20 mM Tris–HCl, 500 mM NaCl and 20 mM imidazole, pH 7.9), followed by elution at a flow rate of 0.3 mL min^−1^ with buffer B as same as buffer A except for 250 mM imidazole. Aliquots of 1 mL eluent containing the target xylanase were pooled, dialyzed against 50 mM Na_2_HPO_4_–citric acid buffer (pH 5.5), and concentrated by ultrafiltration.

### Enzyme activity and protein assays

Xylanase activity was assayed by measuring the amount of reducing sugars from birchwood xylan (Sigma, St. Louis, MO, USA) using the 3,5-dinitrosalicylic acid (DNS) method (Gao et al. 2013). One unit (U) was defined as the amount of xylanase liberating 1 μmol of reducing sugar equivalent per min under the assay conditions (at pH 5.5 and 50 °C for 10 min). The sodium dodecyl sulfate–polyacrylamide gel electrophoresis (SDS-PAGE) was performed according to the method of Laemmli (1970). The separated proteins were visualized by staining with Coomassie Brilliant Blue R-250 (Sigma, USA) and apparent molecular weights of xylanases were estimated by comparison with the standard proteins using the Quantity One software. The protein concentration was measured with a BCA-200 Protein Assay Kit (Pierce, Rockford, IL).

### pH characteristics of the recombinant xylanases

The pH optimum of purified recombinant xylanase was determined under the standard assay conditions, except 5.0 mg mL^−1^ birchwood xylan in 50 mM Na_2_HPO_4_–citric acid buffer at a pH range of 3.0–7.5. Aliquots of xylanase were incubated at 40 °C and varied pH values (Na_2_HPO_4_–citric acid buffer: pH 3.0–7.5; Tris–HCl buffer: pH 8.0–9.0) for 60 min. The pH stability in this work was defined as a pH range, over which the residual xylanase activity retained over 85% of its original one.

### Temperature characteristics of the recombinant xylanases

The temperature optimum (*T*_opt_) of recombinant xylanase was measured, at pH optimum, at temperatures ranging from 40 to 70 °C. The inactivation half-life (*t*_1/2_) of enzyme, a parameter for estimating its thermostability, was defined as a time, when the residual enzyme activity was 50%. In this work, to measure the *t*_1/2_^50^ of recombinant xylanase, aliquots of xylanase were incubated at 50 °C for different times.

The melting temperature (*T*_m_) was defined as a temperature, at which a protein configuration is half unfolded. The protein/enzyme with high *T*_m_ meant it has a high thermostability (Jang et al. 2010). The *T*_m_ of xylanase here was measured by protein thermal shift (PTS) method (Dong et al. 2016), using a PTS Kit (Applied Biosystems, Carlsbad, CA, USA) and a LightCycler 480II 96 Real-Time PCR system (Roche, Basel, Switzerland). Three replicates were conducted independently. The *T*_m_ was a temperature which corresponds to the peak value of a derivative melting curve plotted using the ‘*T*_m_ calling’ method.

### Kinetic parameters of the recombinant xylanases

The reaction rate (U/mg) of birchwood xylan by xylanase was measured under the standard assay conditions, but the substrate concentrations from 1.0 to 10 mg mL^−1^. The reaction rate versus xylan concentration was plotted to verify whether the reaction mode conformed to the Michaelis–Menten equation. The kinetic parameters, *K*_m_ and *k*_cat_, were determined by non-linear regression analysis using an Origin 9.0 software (http://www.originlab.com/). All data were expressed as the mean ± standard deviation (SD) from three independent replicates.

## Results

### Homology modeling of the xylanase 3-D structures

The primary structure identities of AoXyn11A with the three known crystal structure GHF11 xylanases from *P. funiculosum* (PDB code: 1TE1), *T. cellulolyticus* (3WP3) and *E. coli* (2VUL) were 72, 70 and 68%, respectively, suggesting that the crystal structures were suitable as templates. As a result, the 3-D structures of AoXyn11A (Additional file 1: Figure S1) and its variants were homologically modeled, respectively, based on the three templates by SALIGN and MODELLER 9.11 programs, and analyzed by PyMol software.

### Identification of amino acid positions for mutagenesis

**Fig. 1:**
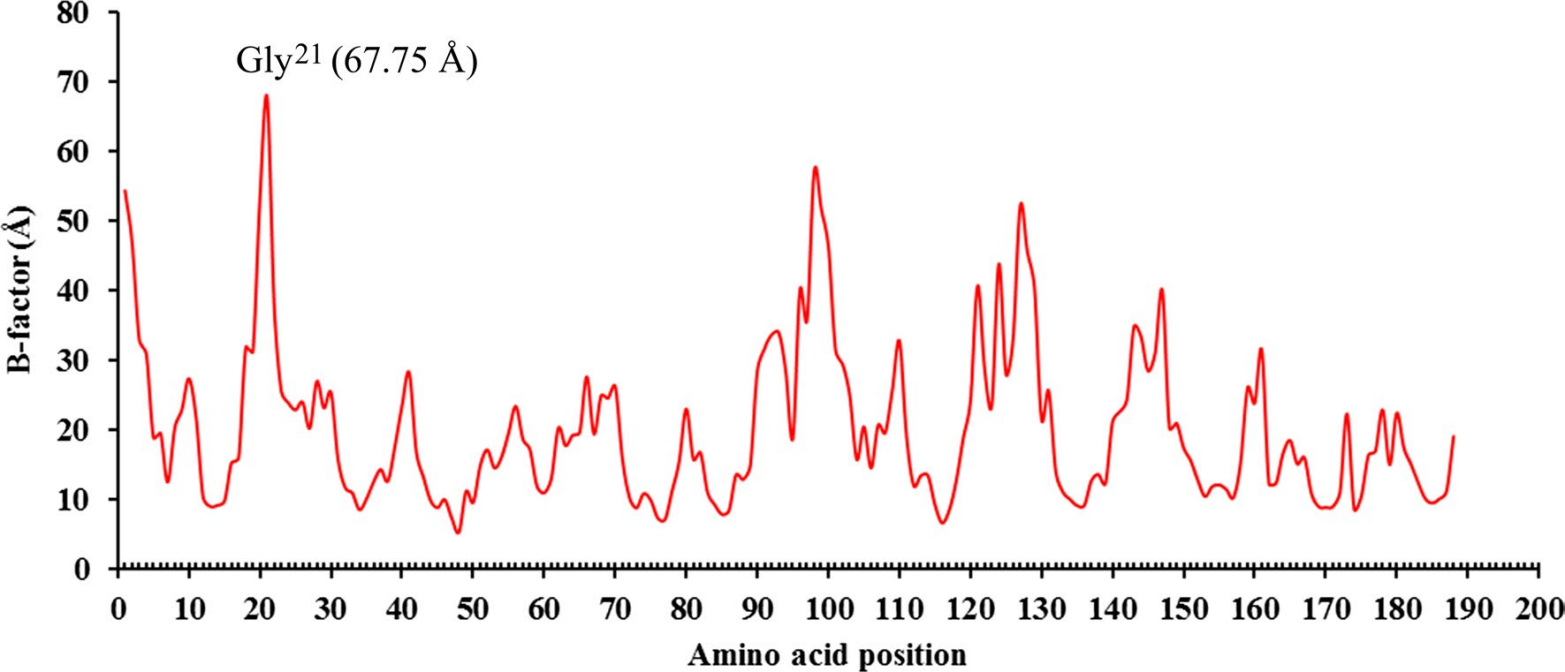
The B-factor values of amino acid residues in AoXyn11A. The modeled 3-D structure of AoXyn11A was subjected to MD simulation at 300 K for 15 ns. Gly^21^ in position 21 with the maximum B-factor value of 67.75 Å was marked

In this work, the B-factor values of amino acid residues in AoXyn11A, generated by MD simulation on its 3-D structure by GROMACS 4.5 package, were calculated using the B-FITTER program (Fig. 1). To improve the thermostability of AoXyn11A, Gly^21^, the amino acid numbered 21, with the maximum B-factor value of 67.75 Å was selected, and then subjected to site-saturation mutagenesis (Yin et al. 2015).

**Fig. 2:**
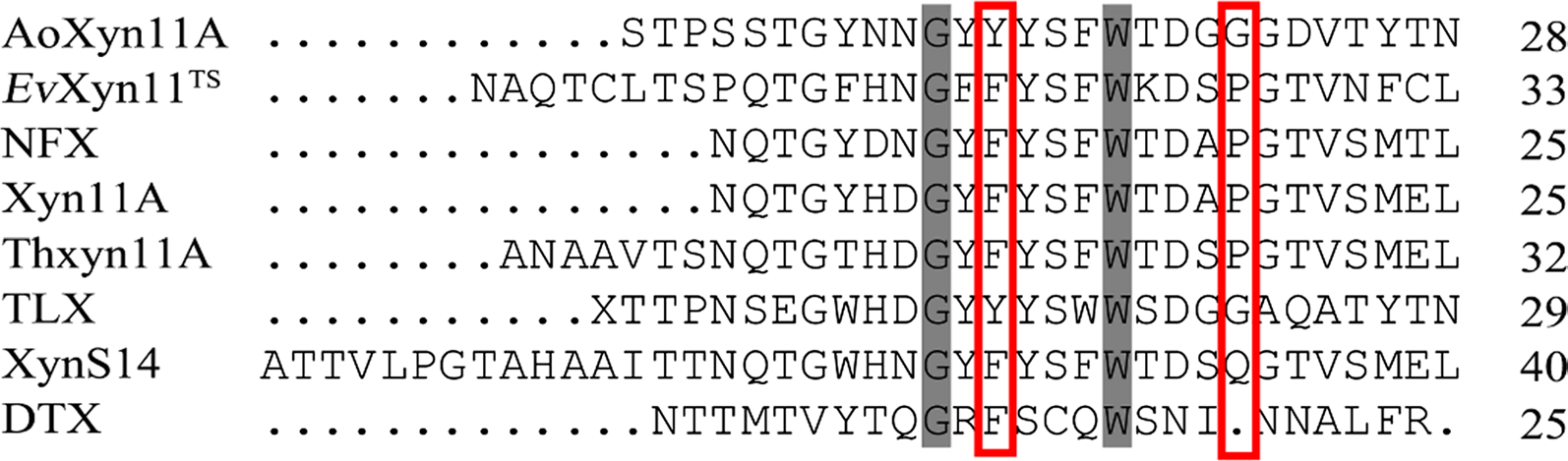
The multiple alignment of the N-terminal amino acid sequences of AoXyn11A and seven thermophilic GHF11 xylanases. The amino acids in positions 13 and 21, numbered by AoXyn11A, are highlighted in *red solid line* frame

**Fig. 3:**
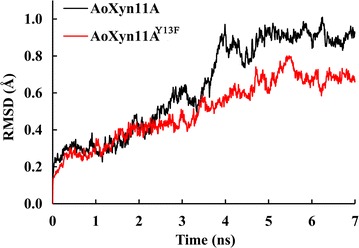
The* curves* of RMSD values of AoXyn11A (*black line*) and AoXyn11A^Y13F^ (*red line*). The modeled 3-D structures of AoXyn11A and AoXyn11A^Y13F^ were subjected to MD simulations, respectively, at 500 K for 7 ns

By consulting the literatures about GHF11 xylanases, the temperature characteristics of seven representative thermophilic GHF11 xylanases, sharing more than 50% identities with that of AoXyn11A, were summarized in Table 1. The multiple alignment of 8 or 11 xylanase primary structures displayed that Tyr^13^ in AoXyn11A (or in AoXyn11A^G21I^) corresponded to Phe in most thermophilic xylanases (Fig. 2; Additional file 1: Figure S2). As a result, the variant xylanase, AoXyn11A^Y13F^ or AoXyn11A^G21I–Y13F^ was designed by substituting Tyr^13^ with Phe. Thereafter, RMSD values of AoXyn11A, AoXyn11A^Y13F^ (Fig. 3) and AoXyn11A^G21I–Y13F^ were calculated. As shown in Fig. 3, the RMSD value of AoXyn11A^Y13F^, after equilibration, was lower than that of AoXyn11A at any time. Thus, the designed AoXyn11A^Y13F^ or AoXyn11A^G21I–Y13F^ was predicted to be more thermostable than AoXyn11A.

**Table 1:** The temperature characteristics of seven representative thermophilic GHF11 xylanases

Xylanase	GenBank no.	Identity (%)	*T*_opt_ (°C)	Thermostability	Reference
*Ev*Xyn11^TS^	ACB87631	63.5	75	*T*_m_ = 101 °C	Dumon et al. (2008)
NFX	CAD48747	56.6	–	*t*_1/2_^100^ = 28 min	Hakulinen et al. (2003)
Xyn11A	AHK22788	56.1	80	80 °C	Zhao et al. (2015)
Thxyn11A	AEH04392	54.7	70	*t*_1/2_^70^ = 90 min	Zhang et al. (2012)
TLX	AAB94633	53.3	75	*t*_1/2_^85^ > 30 min	Hakulinen et al. (2003)
XynS14	BAK09352	52.8	80	*t*_1/2_^80^ = 120 min	Sriyapai et al. (2011)
DTX	AAC46361	52.6	85	–	Hakulinen et al. (2003)

### Screening of the site-saturation mutagenesis library

For the primary screening of the saturation mutagenesis library of Gly^21^, all recombinant xylanases expressed by *E. coli*/*Aoxyn11A* ^G21X^ (X: any amino acid) were treated at 60 °C for 20 min. The results of activity assay and DNA sequencing displayed that seven recombinant variants (Gly^21^ mutated to Phe, Val, Leu, Arg, Tyr, His and Ile, respectively) retained more than 80% of their original activities (Additional file 1: Figure S3), but reAoXyn11A lost its activity completely. Then, four variants (reAoXyn11A^G21F^, reAoXyn11A^G21V^, reAoXyn11A^G21L^ and reAoXyn11A^G21I^) were further screened by extending the treating time to 30 min, whose residual activities were 39.3, 30.5, 37.1 and 57.3%, respectively. Thus, the best strain *E. coli*/*Aoxyn11A* ^G21I^ was selected, expressing reAoXyn11A^G21I^ with the highest thermostability.

### Expression and purification of the recombinant xylanases

It was well known that the expression system of prokaryotes was simple in the gene manipulation and fast in the growth and induction expression rates. Compared to that of prokaryotes, the expression system of eukaryotes had some other advantages, such as extracellular expression, high purity and posttranslational modification, in the production of heterologous proteins/enzymes (Fernández et al. 2010). Based on the comparison of two kinds of expression systems, *E. coli* BL21(DE3) and pET-28a(+) (Novagen, USA) were applied for the construction and screening of a site-saturation mutagenesis library and for the DNA sequencing of a target gene in this work, while *P. pastoris* GS115 and pPIC9K (Invitrogen, USA) used for the expression and purification of recombinant reAoXyn11A and its variants. After *P. pastoris* transformant was induced by methanol for 72 h, the supernatant was used for xylanase activity and protein assays. Among 60 transformants tested, the three ones, labeled as *P. pastoris*/*Aoxyn11A* ^G21I^4-1,/*Aoxyn11A* ^Y13F^4-7 and/*Aoxyn11A* ^G21I–Y13F^4-12 were selected, separately expressing the maximum reAoXyn11A^G21I^, reAoXyn11A^Y13F^ and reAoXyn11A^G21I–Y13F^ activities of 138.8, 89.4 and 79.1 U mL^−1^. Here, *P. pastoris* GS115 transformed with pPIC9K-*Aoxyn11A*, labeled as *P. pastoris*/*Aoxyn11A*, was used as the control.

**Fig. 4:**
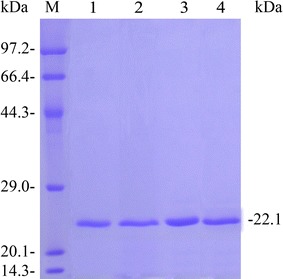
SDS-PAGE analysis of the purified reAoXyn11A and its variants. *Lane M*, protein marker; *lane 1*–*4*, reAoXyn11A, reAoXyn11A^G21I^, reAoXyn11A^Y13F^ and reAoXyn11A^G21I–Y13F^ purified from cultured supernatants, respectively

Three recombinant variants, reAoXyn11A^G21I^, reAoXyn11A^Y13F^ and reAoXyn11A^G21I–Y13F^, were purified to homogeneity by one-step way of Ni–NTA column chromatography. SDS-PAGE analysis of the three recombinant xylanases showed single protein bands with the same apparent molecular weight of about 22.1 kDa (Fig. 4, lanes 2–4). The specific activities of reAoXyn11A^G21I^, reAoXyn11A^Y13F^ and reAoXyn11A^G21I–Y13F^ under the standard assay conditions were 3677, 2280 and 1271 U mg^−1^, respectively, which were 2.3-, 1.4- and 0.8-fold higher than that (1593 U mg^−1^) of reAoXyn11A.

### pH and temperature characteristics of the recombinant xylanases

The purified reAoXyn11A and its three variants exhibited the same pH optimum at pH 5.5 (Additional file 1: Figure S4a). Incubated at 40 °C and varied pH values (3.0–9.0) for 60 min, the four recombinant xylanases were stable over a broad pH range of 4.5–8.5, all retaining more than 85% of their original activities (Additional file 1: Figure S4b).

**Fig. 5:**
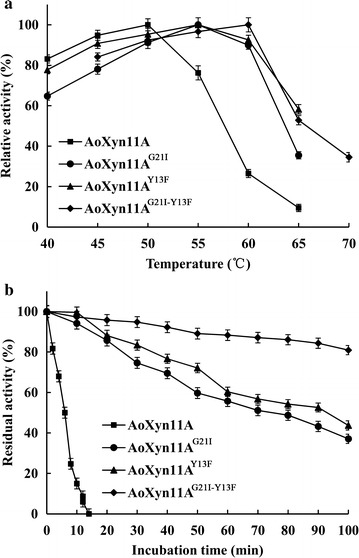
The temperature characteristics of four recombinant xylanases. **a** The *T*_opt_ of xylanase was measured, at pH optimum, at temperatures ranging from 40 to 70 °C. **b** The *t*_1/2_^50^ of xylanase was assayed by incubated it at 50 °C for different times

**Fig. 6:**
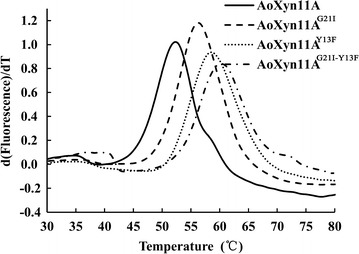
The derivative melting curves of four recombinant xylanases. Emission intensities of fluorescence dye combining with protein hydrophobic regions were recorded from 30 to 80 °C at an elevated rate of 1 °C min^−1^

The *T*_opt_ of reAoXyn11A^G21I–Y13F^ was 60 °C, 5 °C higher than that of reAoXyn11A^G21I^ or reAoXyn11A^Y13F^ and 10 °C higher than that of reAoXyn11A (Fig. 5a). The *t*_1/2_^50^ values of reAoXyn11A^G21I^, AoXyn11A^Y13F^ and reAoXyn11A^G21I–Y13F^ were 71, 95 and 240 min, about 11.8-, 15.8- and 40-fold longer than that of reAoXyn11A (Fig. 5b). The emission intensity of the fluorescence dye combining with protein hydrophobic regions increased as the protein configuration was unfolded at high temperature (Niesen et al. 2007). Based on this mechanism, the *T*_m_ values of reAoXyn11A, reAoXyn11A^G21I^, reAoXyn11A^Y13F^ and reAoXyn11A^G21I–Y13F^ were determined to be 52.3, 56.5, 58.6 and 61.3 °C, respectively (Fig. 6).

### Analysis of the intramolecular interactions in xylanases

**Fig. 7:**
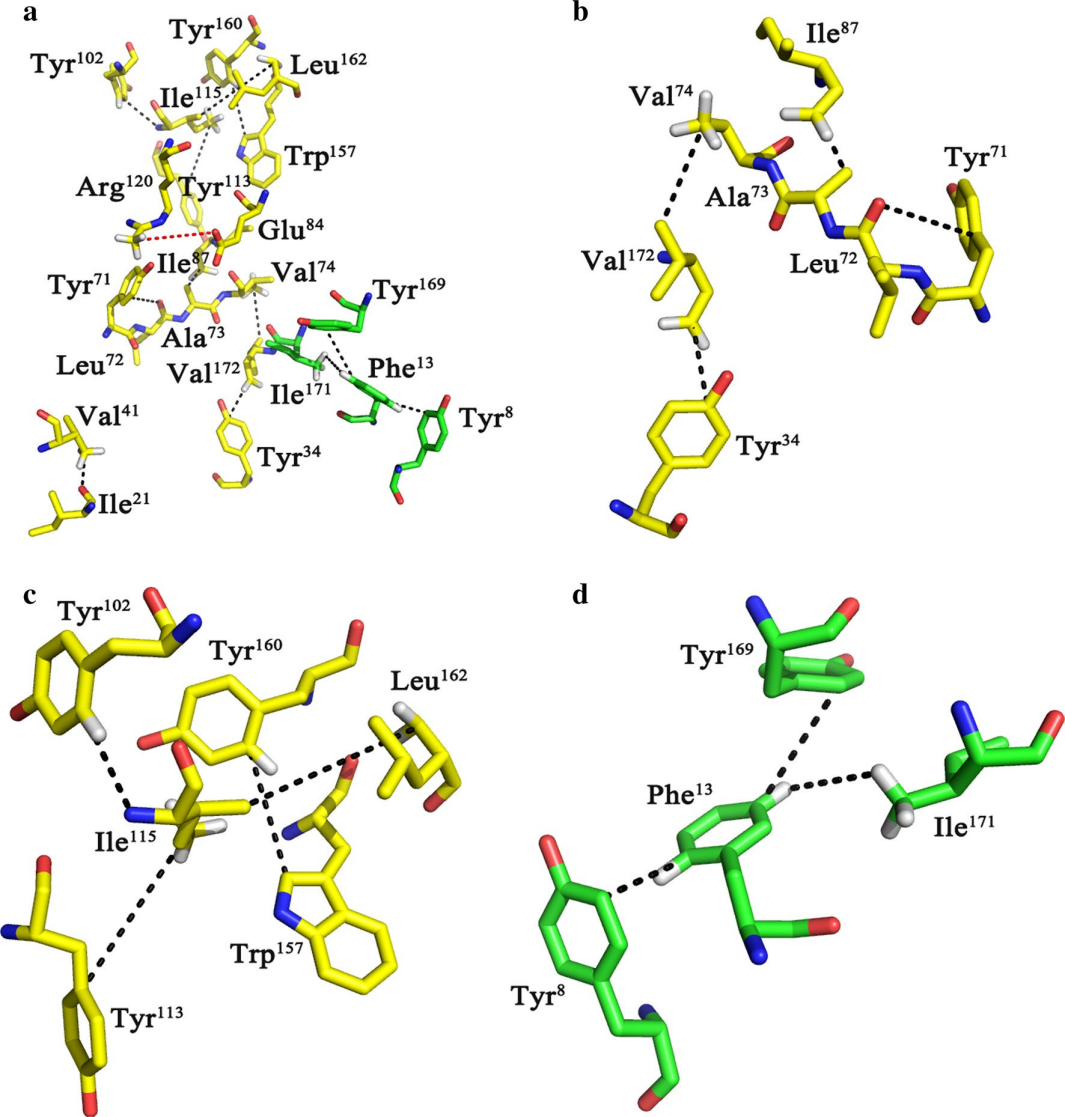
Analysis of the intramolecular interactions in AoXyn11A^G21I–Y13F^. **a** Nine extra hydrophobic interactions (Ile^21^-Val^41^, Tyr^34^-Val^172^, Tyr^71^-Leu^72^, Ala^73^-Ile^87^, Val^74^-Val^172^, Tyr^102^-Ile^115^, Tyr^113^-Ile^115^, Ile^115^-Leu^162^ and Trp^157^-Tyr^160^) and one additional salt bridge (Glu^84^-Arg^120^) were generated by substituting Gly^21^ with Ile. Three extra hydrophobic interactions (Phe^13^-Tyr^8^, Phe^13^-Tyr^169^ and Phe^13^-Ile^171^) were formed by mutation of Tyr13Phe. **b**, **c** Two large hydrophobic regions (Tyr^34^-Tyr^71^-Leu^72^-Ala^73^-Val^74^-Ile^87^-Val^172^ and Tyr^102^-Tyr^113^-Ile^115^-Trp^157^-Tyr^160^-Leu^162^) were illustrated in the locally magnified 3-D structures. **d** Three hydrophobic interactions (Phe^13^-Tyr^8^, Phe^13^-Tyr^169^ and Phe^13^-Ile^171^) were illustrated in the locally magnified 3-D structure

In this work, the differences of intramolecular interactions in AoXyn11A and AoXyn11A^G21I–Y13F^ were analyzed using the PIC server (http://crick.mbu.iisc.ernet.in/PIC). The analytical findings indicated that two hydrophobic interactions (Pro^3^-Phe^16^ and Ile^58^-Val^74^) in AoXyn11A were demolished by substituting Gly^21^ with Ile, but nine additional hydrophobic interactions (Ile^21^-Ver^41^, Tyr^34^-Val^172^, Tyr^71^-Leu^72^, Ala^73^-Ile^87^, Val^74^-Val^172^, Tyr^102^-Ile^115^, Tyr^113^-Ile^115^, Ile^115^-Leu^162^ and Trp^157^-Tyr^160^) and one extra salt bridge (Glu^84^-Arg^120^) in AoXyn11A^G21I–Y13F^ were generated (Fig. 7a). The mutation of Gly21Ile produced two large hydrophobic regions (Tyr^34^-Tyr^71^-Leu^72^-Ala^73^-Val^74^-Ile^87^-Val^172^ and Tyr^102^-Tyr^113^-Ile^115^-Trp^157^-Tyr^160^-Leu^162^) (Fig. 7b, c). The hydrophobic parameters of amino acids Tyr and Phe, which were positively correlative to their hydrophobicities, were -1.3 and 2.8, respectively (Thorton and Taylor 1989). Thus, it was confirmed that the hydrophobicity of Phe was more powerful than that of Tyr. Owing to the substitution of Tyr^13^ with Phe, three additional hydrophobic interactions (Phe^13^-Tyr^8^, Phe^13^-Tyr^169^ and Phe^13^-Ile^171^) presented in AoXyn11A^G21I–Y13F^ were generated (Fig. 7d). These intramolecular interactions in the variants, AoXyn11A^G21I^, AoXyn11A^Y13F^ and AoXyn11A^G21I–Y13F^, played important roles in their improved temperature characteristics.

### Kinetic parameters of the recombinant xylanases

The reaction mode of reAoXyn11A or its any recombinant variant conformed to the Michaelis–Menten equation. *K*_m_ and *k*_cat_ values of four recombinant xylanases towards birchwood xylan were determined and listed in Table 2. Compared with *K*_m_ value (3.33 mg mL^−1^) of reAoXyn11A, those of reAoXyn11A^G21I^ and reAoXyn11A^G21I–Y13F^ were decreased by 30.3 and 37.5%, respectively, while that of reAoXyn11A^Y13F^ slightly increased by 4.2%. The *k*_cat_ values of reAoXyn11A, reAoXyn11A^G21I^, reAoXyn11A^Y13F^ and reAoXyn11A^G21I–Y13F^ were 954.1, 1246.8, 1137.5 and 984.1 s^−1^. Consequently, the catalytic efficiency, *k*_cat_/*K*_m_, of reAoXyn11A^G21I–Y13F^ (473.1 mL mg^−1^ s^−1^) was higher than those (286.5 and 327.8 mL mg^−1^ s^−1^) of reAoXyn11A and reAoXyn11A^Y13F^, but lower than that (537.4 mL mg^−1^ s^−1^) of reAoXyn11A^G21I^.

**Table 2:** The kinetic parameters of reAoXyn11A and its three variants

Recombinant xylanase	*K*_m_ (mg mL^−1^)	*k*_cat_ (s^−1^)	*k*_cat_/*K*_m_ (mL mg^−1^ s^−1^)	Fold
reAoXyn11A	3.33 ± 0.2	954.1 ± 49	286.5	1.00
reAoXyn11A^G21I^	2.32 ± 0.1	1246.8 ± 67	537.4	1.88
reAoXyn11A^Y13F^	3.47 ± 0.2	1137.5 ± 53	327.8	1.14
reAoXyn11A^G21I–Y13F^	2.08 ± 0.1	984.1 ± 40	473.1	1.65

*K*_m_ and *k*_cat_ values were measured under the standard assay conditions, except birchwood xylan concentrations from 1.0 to 10 mg mL^−1^. Data were expressed as the mean ± standard deviation (SD) from three independent replicates

## Discussion

Based on the permutation and combination of three continuous nucleotides or bases, the degenerate triad NNK (N: A/C/G/T; K: G/T) can constitute 32 codons (containing a terminator codon of TAG), which can encode all kinds of 20 amino acids. Therefore, NNK was commonly used for the site-saturation mutagenesis of a specific amino acid (Tian et al. 2013). The number of single colonies that should be screened for 95% coverage in the case of randomization at one amino acid position was 94, which could be predicted using the CASTER program (Yin et al. 2015). Therefore, more than 94 single colonies in this work were picked out from the site-saturation mutagenesis library of Gly^21^ in AoXyn11A (*E. coli*/*Aoxyn11A* ^G21X^, X: any one of 20 residues) to select the best strain, which expressing the recombinant variant xylanase with the highest thermostability. Generally, based on their respective advantages, the prokaryote expression system was applied for the construction and screening of mutagenesis libraries, while the eukaryote one used for the production of recombinant proteins/enzymes.

For the site-directed and site-saturation mutagenesis, the identification of the amino acid residue sites or the substituted residues was a very inconvenient and time-consuming process. Fortunately, to date, a large amount of bioinformation about the relationship between protein’s structure and function has been calculated and analyzed by means of computer programs and softwares. Based on the computer-aided design, the key residues and their sites in the primary and 3-D structures, the substituted residues, and some properties of proteins/enzymes could be predicted more accurately and quickly (Lassila 2010). For example, some determinants for the improved thermostability of a mesophilic GHF11 xylanase, AuXyn11A, were predicted using the computational methods (Zhang et al. 2014). In this work, based on the 3-D structure homology modeling, MD simulation and B-factor value calculation, one specific amino acid and its position, namely Gly^21^, in AoXyn11A was confirmed for the site-saturation mutagenesis of Gly21X (X: any one of 20 amino acids) in AoXyn11A. Furthermore, based on the primary structure multiple alignment of AoXyn11A (or AoXyn11A^G21I^) with seven representative thermophilic GHF11 xylanases, and on the calculation and comparison of RMSD values of AoXyn11A and its variants, Tyr^13^ in AoXyn11A or AoXyn11A^G21I^ was identified for the site-directed mutagenesis of Tyr13Phe.

Both amino acid residues, Tyr^13^ and Gly^21^, in AoXyn11A are located at its N-terminal region. Based on the modeling and analysis of the 3-D structure of AoXyn11A, it was identified that Tyr^13^ is located in the β-strand B2, while Gly^21^ lies in the loop between the β-strands B2 and A2 (Additional file 1: Figure S1). Unlike the C-terminus of GHF11 xylanase, which is buried in the center of two β-sheets A and B, its N-terminus lies in the fringe of two β-sheets and is exposed to the hydrophilic environment. Deduced from the above analytical results, the N-terminus is denatured more easily than the C-terminus at high temperature. The denaturation of GHF11 xylanase originated from its N-terminus, which would result in the disassembly of the whole 3-D structure of protein (Li et al. 2012). In our previous studies, N-terminus modifications of AoXyn11A were performed to improve its thermostability by different routes. The *T*_opt_ and *t*_1/2_^70^ of reAEx11A, a hybrid xylanase constructed by substituting the N-terminus of AoXyn11A with the corresponding region of *Ev*Xyn11^TS^, was 30 °C higher and 197-fold longer than those of reAoXyn11A. But the *V*_max_ of reAEx11A decreased to 804.4 μmol min^−1^ mg^−1^ (Gao et al. 2013). It was verified that the disulfide bridge in the N-terminus of AEx11A mainly contributed to its high thermostability (Tan et al. 2014). Besides, the *T*_opt_ of reAoXyn11A^C5/C32^ was increased 10 °C by introducing a disulfide bridge between the β-strands A1 and A2 of AoXyn11A (Chen et al. 2013). In this work, both the temperature characteristics and catalytic efficiency of AoXyn11A were obviously improved by iterative mutagenesis of Gly21Ile and Tyr13Phe in the N-terminus.

Some minor or local modifications in the primary and 3-D structures related to the improved thermostability of GHF11 xylanases have been extensively reported, such as the increased number of charged residues, ion pairs and aromatic residues on the protein surface as well as the insertions to certain regions resulting in enhanced intramolecular interactions (You et al. 2010). These modifications or changes greatly increased the rigidity of a protein configuration, the property associated with its enhanced thermostability. Reportedly, several variants of *Bacillus circulans* xylanase with strengthened hydrophobic interactions in some local 3-D structures were much more thermostable than a wild-type xylanase (Joo et al. 2011). Similarly, the thermostability of xylanase XynR8 from *Neocallimastigales rumen fungal* was significantly improved by introducing several salt bridges into it (Xue et al. 2012). In this work, the structural analysis indicated that the additional hydrophobic interactions and a salt bridge in AoXyn11A^G21I–Y13F^ make its N-terminus and whole 3-D structure more stable at high temperature, thereby obviously improving the thermostability of AoXyn11A.

In conclusion, based on the computer-aided design, the temperature characteristics and catalytic efficiency of a wild-type AoXyn11A were remarkably improved by site-saturation mutagenesis and iterative mutagenesis, making reAoXyn11A^G21I–Y13F^ a promising candidate for industrial bioprocesses where the high temperature was required. This work also provided an effective strategy for the directed modification of other GHF11 xylanases, especially those from fungi, to perfect their enzymatic properties.

## Additional file

**Additional file 1: Table S1.** PCR primers used for the site-saturation and site-directed mutagenesis. **Figure S1.** The modeled 3-D structure of AoXyn11A. **Figure S2.** The primary structure multiple alignment of AoXyn11A with three homology modeling templates and seven thermophilic GHF11 xylanases. **Figure S3.** The primary screening of the site-saturation mutagenesis library of Gly^21^ in AoXyn11A. **Figure S4.** The pH characteristics of our recombinant xylanases.

## Abbreviations

GHF11: glycoside hydrolase family 11
3-D: three-dimensional
AoXyn11A: GHF11 xylanase from *Aspergillus oryzae*
*Aoxyn11A*: AoXyn11A gene
PDB: Protein Data Bank
MD: molecular dynamics
RMSD: root mean square deviation
IPTG: isopropyl β-d-1-thiogalactopyranoside
Ni–NTA: nickel–nitrilotriacetic acid
SDS-PAGE: sodium dodecyl sulfate–polyacrylamide gel electrophoresis
DNS: 3,5-dinitrosalicylic acid
*T*_opt_: temperature optimum
*t*_1/2_: thermal inactivation half-life
*T*_m_: melting temperature
PTS: protein thermal shift
*k*_cat_/*K*_m_: catalytic efficiency

## References

[CR1] Badieyan S, Bevan DR, Zhang C (2012). Study and design of stability in GH5 cellulases. Biotechnol Bioeng.

[CR2] Balaa BA, Brijs K, Gebruers K, Vandenhaute J, Wouters J, Housen I (2009). Xylanase XYL1p from *Scytalidium acidophilum*: site-directed mutagenesis and acidophilic adaptation. Bioresour Technol.

[CR3] Chen ZF, Zhang HM, Wang JQ, Tang CD, Wu J, Wu MC (2013). Engineering the thermostability of a xylanase from *Aspergillus oryzae* by an enhancement of the interactions between the N-terminus extension and the β-sheet A2 of the enzyme. Biotechnol Lett.

[CR4] Dong YH, Li JF, Hu D, Yin X, Wang CJ, Tang SH, Wu MC (2016). Replacing a piece of loop-structure in the substrate-binding groove of *Aspergillus usamii* β-mannanase, AuMan5A, to improve its enzymatic properties by rational design. Appl Microbiol Biotechnol.

[CR5] Dumon C, Varvak A, Wall MA, Flint JE, Lewis RJ, Lakey JH, Morland C, Luginbühl P, Healey S, Todaro T, DeSantis G, Sun M, Parra-Gessert L, Tan X, Weiner DP, Gilbert HJ (2008). Engineering hyperthermostability into a GH11 xylanase is mediated by subtle changes to protein structure. J Biol Chem.

[CR6] Fernández L, Jiao N, Soni P, Gumulya Y, de Oliveira LG, Reetz MT (2010). An efficient method for mutant library creation in *Pichia pastoris* useful in directed evolution. Biocatal Biotransfor.

[CR7] Gao SJ, Wang JQ, Wu MC, Zhang HM, Yin X, Li JF (2013). Engineering hyperthermostability into a mesophilic family 11 xylanase from *Aspergillus oryzae* by in silico design of N-terminus substitution. Biotechnol Bioeng.

[CR8] Hakulinen N, Turunen O, Jӓnis J, Leisola M, Rouvinen J (2003). Three-dimensional structures of thermophilic β-1,4-xylanases from *Chaetomium thermophilum* and *Nonomuraea flexuosa*: comparison of twelve xylanases in relation to their thermal stability. Eur J Biochem.

[CR9] Irfan M, Guler HI, Ozer A, Sapmaz MT, Belduz AO, Hasan F, Shah AA (2016). C-Terminal proline-rich sequence broadens the optimal temperature and pH ranges of recombinant xylanase from *Geobacillus thermodenitrificans* C5. Enzyme Microb Technol.

[CR10] Jaenicke R, Böhm G (1998). The stability of proteins in extreme environments. Curr Opin Struct Biol.

[CR11] Jang MK, Lee SW, Lee DG, Kim NY, Yu KH, Jang HJ, Kim S, Kim A, Lee SH (2010). Enhancement of the thermostability of a recombinant β-agarase, AgaB, from *Zobellia galactanivorans* by random mutagenesis. Biotechnol Lett.

[CR12] Joo JC, Pack SP, Kim YH, Yoo YJ (2011). Thermostabilization of *Bacillus circulans* xylanase: computational optimization of unstable residues based on thermal fluctuation analysis. J Biotechnol.

[CR13] Kataoka M, Akita F, Maeno Y, Inoue B, Inoue H, Ishikawa K (2014). Crystal structure of *Talaromyces cellulolyticus* (formerly known as *Acremonium cellulolyticus*) GH family 11 xylanase. Appl Biochem Biotechnol.

[CR14] Kumar V, Marín-Navarro J, Shukla P (2016). Thermostable microbial xylanases for pulp and paper industries: trends, applications and further perspectives. World J Microbiol Biotechnol.

[CR15] Laemmli UK (1970). Cleavage of structural proteins during the assembly of the head of bacteriophage T4. Nature.

[CR16] Lassila JK (2010). Conformational diversity and computational enzyme design. Curr Opin Chem Biol.

[CR17] Li H, Murtomäki L, Leisola M, Turunen O (2012). The effect of thermostabilising mutations on the pressure stability of *Trichoderma reesei* GH11 xylanase. Protein Eng Des Sel.

[CR18] Li JF, Gao SJ, Liu XT, Gong YY, Chen ZF, Wei XH, Zhang HM, Wu MC (2013). Modified pPIC9K vector-mediated expression of a family 11 xylanase gene, *Aoxyn11A*, from *Aspergillus oryzae* in *Pichia pastoris*. Ann Microbiol.

[CR19] Luo HY, Yang J, Li J, Shi PJ, Huang HQ, Bai YG, Fan YL, Yao B (2010). Molecular cloning and characterization of the novel acidic xylanase XYLD from *Bispora* sp. MEY-1 that is homologous to family 30 glycosyl hydrolases. Appl Microbiol Biotechnol.

[CR20] Madhusudhan MS, Webb BM, Marti-Renom MA, Eswar N, Sali A (2009). Alignment of multiple protein structures based on sequence and structure features. Protein Eng Des Sel.

[CR21] Niesen FH, Berglund H, Vedadi M (2007). The use of differential scanning fluorimetry to detect ligand interactions that promote protein stability. Nat Protoc.

[CR22] Payan F, Leone P, Porciero S, Furniss C, Tahir T, Williamson G, Durand A, Manzanares P, Gilbert HJ, Juge N, Roussel A (2004). The dual nature of the wheat xylanase protein inhibitor XIP-I: structural basis for the inhibition of family 10 and family 11 xylanases. J Biol Chem.

[CR23] Reetz MT, Carballeira JD (2007). Iterative saturation mutagenesis (ISM) for rapid directed evolution of functional enzymes. Nat Protoc.

[CR24] Sanchis J, Fernández L, Carballeira JD, Drone J, Gumulya Y, Höbenreich H, Kahakeaw D, Kille S, Lohmer R, Peyralans JJ, Podtetenieff J, Prasad S, Soni P, Taglieber A, Wu S, Zilly FE, Reetz MT (2008). Improved PCR method for the creation of saturation mutagenesis libraries in directed evolution: application to difficult-to-amplify templates. Appl Microbiol Biotechnol.

[CR25] Silva IR, Larsen DM, Jers C, Derkx P, Meyer AS, Mikkelsen JD (2013). Enhancing RGI lyase thermostability by targeted single point mutations. Appl Microbiol Biotechnol.

[CR26] Sokkar P, Mohandass S, Ramachandran M (2011). Multiple templates-based homology modeling enhances structure quality of AT1 receptor: validation by molecular dynamics and antagonist docking. J Mol Model.

[CR27] Song L, Dumon C, Siguier B, Andre I, Eneyskaya E, Kulminskaya A, Bozonnet S, O’Donohue MJ (2014). Impact of an N-terminal extension on the stability and activity of the GH11 xylanase from *Thermobacillus xylanilyticus*. J Biotechnol.

[CR28] Sriyapai T, Somyoonsap P, Matsui K, Kawai F, Chansiri K (2011). Cloning of a thermostable xylanase from *Actinomadura* sp. S14 and its expression in *Escherichia coli* and *Pichia pastoris*. J Biosci Bioeng.

[CR29] Stephens DE, Khan FI, Singh P, Bisetty K, Singh S, Permaul K (2014). Creation of thermostable and alkaline stable xylanase variants by DNA shuffling. J Biotechnol.

[CR30] Tan ZB, Tang CD, Wu MC, He Y, Hu D, Wang JQ (2014). Exploration of disulfide bridge and *N*-glycosylation contributing to high thermostability of a hybrid xylanase. Protein Pept Lett.

[CR31] Thorton J, Taylor WR, Findlay JBC, Geisow MJ (1989). Structure prediction. The IRL Press series in protein sequencing.

[CR32] Tian J, Wang P, Huang L, Chu XY, Wu NF, Fan YL (2013). Improving the thermostability of methyl parathion hydrolase from *Ochrobactrum* sp. M231 using a computationally aided method. Appl Microbiol Biotechnol.

[CR33] Trevizano LM, Ventorim RZ, de Rezende ST, Junior FPS, Guimarães VM (2012). Thermostability improvement of *Orpinomyces* sp. xylanase by directed evolution. J Mol Catal B Enzym.

[CR34] Watanabe M, Fukada H, Ishikawa K (2016). Construction of thermophilic xylanase and its structural analysis. Biochemistry.

[CR35] Xue HP, Zhou JG, You C, Huang Q, Lu H (2012). Amino acid substitutions in the N-terminus, cord and α-helix domains improved the thermostability of a family 11 xylanase XynR8. J Ind Microbiol Biotechnol.

[CR36] Yin X, Li JF, Wang CJ, Hu D, Wu Q, Gu Y, Wu MC (2015). Improvement in the thermostability of a type A feruloyl esterase, AuFaeA, from *Aspergillus usamii* by iterative saturation mutagenesis. Appl Microbiol Biotechnol.

[CR37] You C, Huang Q, Xue H, Xu Y, Lu H (2010). Potential hydrophobic interaction between two cysteines in interior hydrophobic region improves thermostability of a family 11 xylanase from *Neocallimastix patriciarum*. Biotechnol Bioeng.

[CR38] Zhang F, Chen JJ, Ren WZ, Lin LB, Zhou Y, Zhi XY, Tang SK, Li WJ (2012). Cloning, expression, and characterization of an alkaline thermostable GH11 xylanase from *Thermobifida halotolerans* YIM 90462^T^. J Ind Microbiol Biotechnol.

[CR39] Zhang HM, Li JF, Wang JQ, Yang YJ, Wu MC (2014). Determinants for the improved thermostability of a mesophilic family 11 xylanase predicted by computational methods. Biotechnol Biofuels.

[CR40] Zhao LM, Geng J, Guo YQ, Liao XD, Liu XH, Wu RJ, Zheng ZJ, Zhang RJ (2015). Expression of the *Thermobifida fusca* xylanase Xyn11A in *Pichia pastoris* and its characterization. BMC Biotechnol.

[CR41] Zheng HC, Liu YH, Sun MZ, Han Y, Wang JL, Sun JS, Lu FP (2014). Improvement of alkali stability and thermostability of *Paenibacillus campinasensis* family-11 xylanase by directed evolution and site-directed mutagenesis. J Ind Microbiol Biotechnol.

