# Supplementary material for: Improving the temperature characteristics and catalytic efficiency of a mesophilic xylanase from Aspergillus oryzae, AoXyn11A, by iterative mutagenesis based on in silico design
Source: AMB Express. 2017 May 15;7:97. doi: 10.1186/s13568-017-0399-9 (PMC5432455; doi:10.1186/s13568-017-0399-9)
Supplement: Supplementary file 1 — Additional file 1: Table S1. PCR primers used for the site-saturation and site-directed mutagenesis. Figure S1. The modeled 3-D structure of AoXyn11A. Figure S2. The primary structure multiple alignment of AoXyn11A with three homology modeling templates and seven thermophilic GHF11 xylanases. Figure S3. The primary screening of the site-saturation mutagenesis library of Gly21 in AoXyn11A. Figure S4. The pH characteristics of our recombinant xylanases. [file 13568_2017_399_MOESM1_ESM.pdf]

AMB Express

Improving the temperature characteristics and catalytic efficiency of a mesophilic xylanase from *Aspergillus oryzae*, AoXyn11A, by iterative mutagenesis based on in silico design

Xue-Qing Li[1] ·Qin Wu[2] ·Die Hu[2] ·Rui Wang[2] ·Yan Liu[1] ·Min-Chen Wu[3] ·Jian-Fang Li[1]

[1] School of Food Science and Technology, Jiangnan University, 1800 Lihu Road, Wuxi 214122, China

Xue-Qing Li: 2116619184@qq.com; Yan Liu: 1203767169@qq.com; Jian-Fang Li* (✉): lijf@163.com

[2] Key Laboratory of Carbohydrate Chemistry and Biotechnology, Ministry of Education, School of Biotechnology, Jiangnan University, 1800 Lihu Road, Wuxi 214122, China

Qin Wu: wuqinzy@163.com; Die Hu: buttflystudy@163.com; Rui Wang: 1004906850@qq.com

[3] Wuxi Medical School, Jiangnan University, 1800 Lihu Road, Wuxi 214122, China

Min-Chen Wu* (✉): biowmc@126.com

Xue-Qing Li and Qin Wu, the two first authors, contributed equally to this work.

* Corresponding authors.

Supplementary information

Table S1 PCR primers used for the site-saturation and site-directed mutagenesis.

Fig. S1 The modeled 3-D structure of AoXyn11A.

Fig. S2 The primary structure multiple alignment of AoXyn11A with three homology modeling templates and seven thermophilic GHF11 xylanases.

Fig. S3 The primary screening of the site-saturation mutagenesis library of Gly^{21} in AoXyn11A.

Fig. S4 The pH characteristics of four recombinant xylanases.

Table S1 PCR primers used for the site-saturation and site-directed mutagenesis

Primer name	Primer sequence (5′–3′)	Size (bp)
G21X-F (Forward)	TTCTGGACCGACGGCNNKGGTGATGTGACTTAC	33
X11-R (Reverse)	GCGGCCGCTCAATAAACAGTGATAGCAG	28
Y13F-F (Forward)	TATAACAATGGCTACTTCTACTCCTTCTGGACC	33

The framed NNK (N: A/C/G/T; K: G/T) was used for the site-saturation mutagenesis of Gly^{21} in AoXyn11A, while the framed TTC used for the site-directed mutagenesis of Tyr^{13} in AoXyn11A or AoXyn11A^{G21I} into Phe. The underlined base sequence indicates a *Not* Ⅰ site.

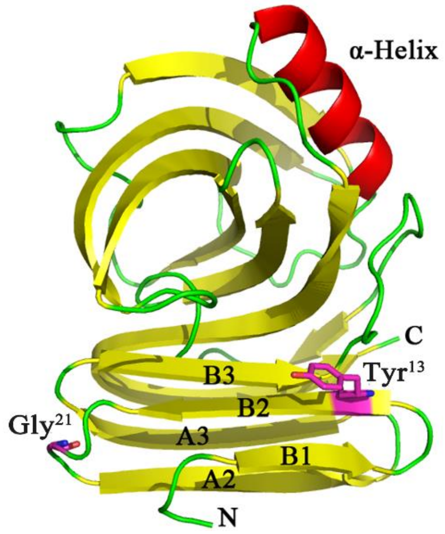

Fig. S1 The modeled 3-D structure of AoXyn11A. Tyr^{13} is located in the β-strand B2, while Gly^{21} in the loop between the β-strands B2 and A2

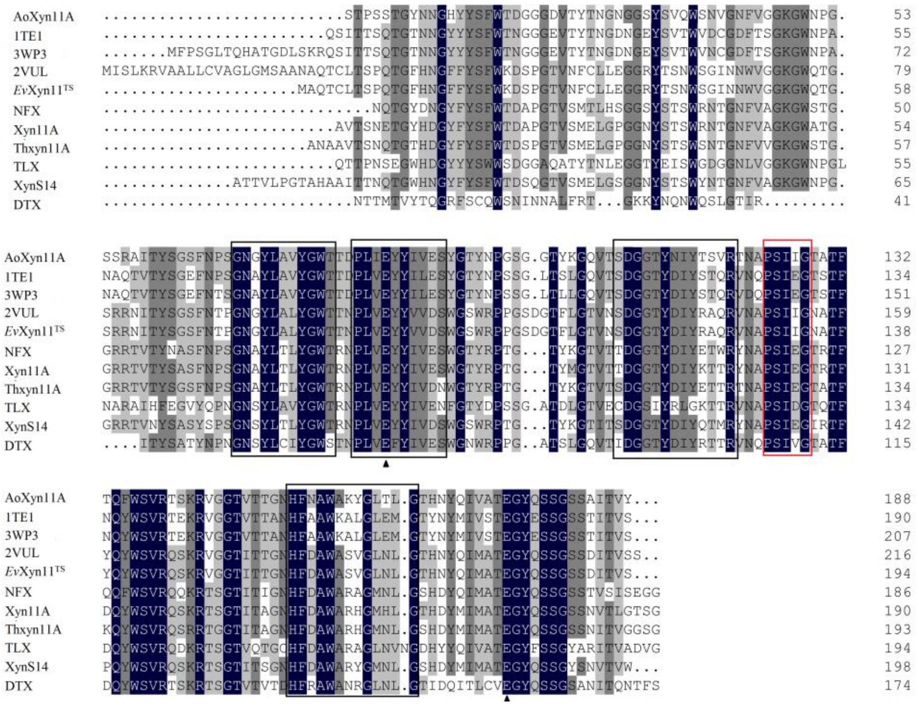

Fig. S2 The primary structure multiple alignment of AoXyn11A with three homology modeling templates and seven thermophilic GHF11 xylanases. The four most conserved motifs (NGYLSVYGWT, PLIEYYIVES, SDGSVYDIYTATR and HFNAWAKLGMNLG) in all GHF11 xylanases are marked in black solid line frame. One conserved sequence PSIXG (X: any residue) in the 'thumb' is marked in red solid line frame. The two triangles indicate two catalytic residues (acid/base, Glu^{84} and nucleophile, Glu^{175}, numbered by AoXyn11A)

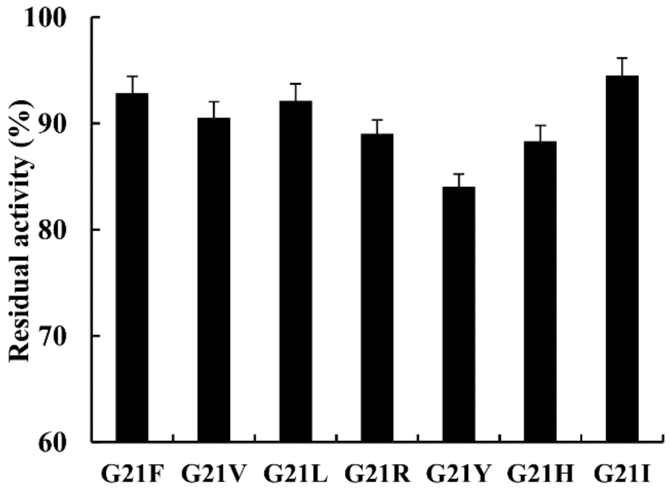

Fig. S3 The primary screening of the site-saturation mutagenesis library of Gly^{21} in AoXyn11A. The seven recombinant variants retained over 80 % of their original activities after treated at 60 ℃ for 20 min

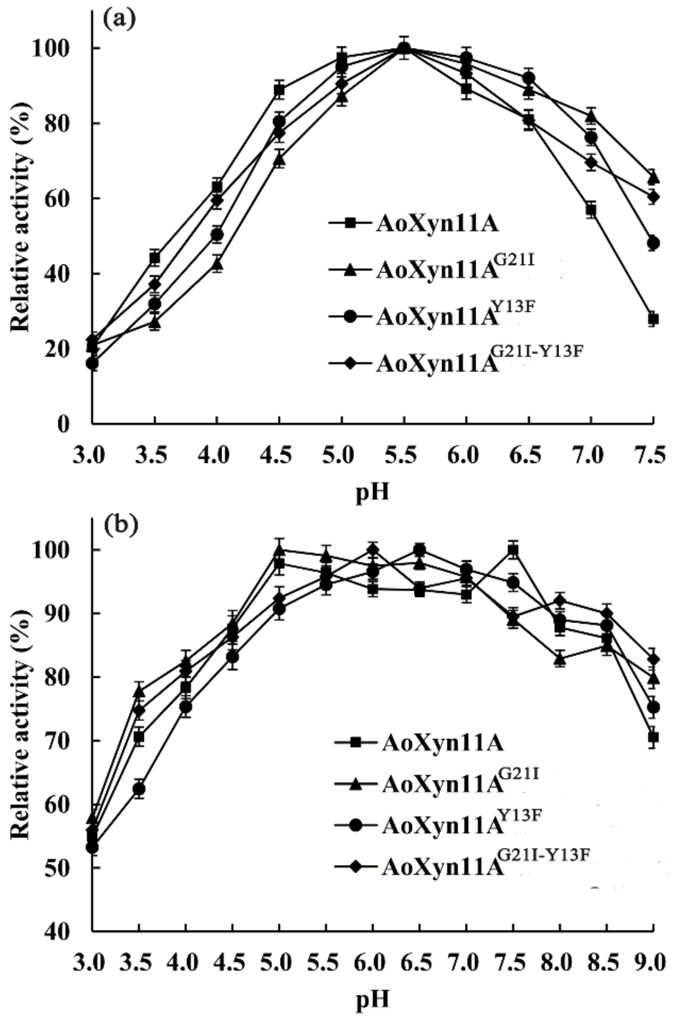

Fig. S4 The pH characteristics of four recombinant xylanases. **a** The pH optimum of xylanase was assayed under the standard assay conditions, except for pH values ranging from 3.0 to 7.5. **b** The pH stability of xylanase was measured by incubated it at 40 ℃ and varied pH values (3.0–9.0) for 60 min
